# Correlation of Electrocardiographic Changes with Cardiac Magnetic Resonance Findings in Patients with Hypertrophic Cardiomyopathy

**DOI:** 10.5935/abc.20170189

**Published:** 2018-01

**Authors:** Gabriela Miana de Mattos Paixão, Horácio Eduardo Veronesi, Halsted Alarcão Gomes Pereira da Silva, José Nunes de Alencar Neto, Carolina de Paulo Maldi, Luciano de Figueiredo Aguiar Filho, Ibrahim Masciarelli Francisco Pinto, Francisco Faustino de Albuquerque Carneiro de França, Edileide de Barros Correia

**Affiliations:** Instituto Dante Pazzanese de Cardiologia, São Paulo, SP - Brazil

**Keywords:** Hypertrophic cardiomyopathy / genetic, Electrocardiography, Magnetic Resonance Spectroscopy

## Abstract

**Background:**

Electrocardiogram is the initial test in the investigation of heart disease. Electrocardiographic changes in hypertrophic cardiomyopathy have no set pattern, and correlates poorly with echocardiographic findings. Cardiac magnetic resonance imaging has been gaining momentum for better assessment of hypertrophy, as well as the detection of myocardial fibrosis.

**Objectives:**

To correlate the electrocardiographic changes with the location of hypertrophy in hypertrophic cardiomyopathy by cardiac magnetic resonance.

**Methods:**

This descriptive cross-sectional study evaluated 68 patients with confirmed diagnosis of hypertrophic cardiomyopathy by cardiac magnetic resonance. The patients’ electrocardiogram was compared with the location of the greatest myocardial hypertrophy by cardiac magnetic resonance. Statistical significance level of 5% and 95% confidence interval were adopted.

**Results:**

Of 68 patients, 69% had septal hypertrophy, 21% concentric and 10% apical hypertrophies. Concentric hypertrophy showed the greatest myocardial fibrosis mass (p < 0.001) and the greatest R wave size in D1 (p = 0.0280). The amplitudes of R waves in V5 and V6 (p = 0.0391, p = 0.0148) were higher in apical hypertrophy, with statistical significance. Apical hypertrophy was also associated with higher T wave negativity in D1, V5 and V6 (p < 0.001). Strain pattern was found in 100% of the patients with apical hypertrophy (p < 0.001).

**Conclusion:**

The location of myocardial hypertrophy by cardiac magnetic resonance can be correlated with electrocardiographic changes, especially for apical hypertrophy.

## Introduction

Hypertrophic cardiomyopathy (HCM) is an autosomal dominant disease, characterized by myocardial hypertrophy in the absence of cardiac or systemic diseases.^1^ In adults, the diagnosis is defined by a diastolic thickness of any ventricular wall ≥ 15 mm measured on any imaging test.^2^

Electrocardiogram (ECG) is the initial test to be performed when investigating heart diseases. In HCM, the ECG has not a defined pattern, and can show signs of left ventricular overload, presence of q waves, changes in the ST segment, abnormal T waves, or be even normal in 6% of the patients.^3^

Some specific electrocardiographic findings may suggest the location of hypertrophy, as well as the presence of fibrosis. Giant inverted T waves (> 10 mm) in the precordial or inferolateral leads suggest apical involvement. Deep q waves in the inferolateral leads with positive T waves are associated with the asymmetric distribution of the HCM, and q waves lasting more than 40 ms relate to fibrosis. The ST-segment elevation in the lateral wall can correlate to small apical aneurysms, which are associated with fibrosis.^2^ Electrocardiographic patterns similar to that of myocardial infarction in young individuals can precede the echocardiographic evidence of myocardial hypertrophy.^4^

The electrocardiographic changes can lead to the suspicion of HCM, and together with the clinical history and other imaging tests can establish the diagnosis.

Traditionally, echocardiography is the imaging test of choice to diagnose HCM, because of its wide availability and lower cost. However, the relationship between the electrocardiographic changes and the morphology and severity of myocardial thickness has not been well established when assessed on the echocardiogram.^5,6^

Cardiac magnetic resonance imaging has gained importance in the HCM assessment, because of its superiority in measuring the thickness of ventricular walls, mainly in regions difficult to visualize on echocardiography, in addition to providing ventricular morphology and function assessment. Detection of delayed contrast-enhancement has prognostic value.^2^

This study was aimed at correlating electrocardiographic variables with the location of myocardial hypertrophy assessed on cardiac magnetic resonance imaging.

## Methods

This is a descriptive cross-sectional study that assessed patients diagnosed with HCM and followed up at the Cardiomyopathy Sector of the Instituto Dante Pazzanese de Cardiologia (IDPC), who underwent cardiac magnetic resonance imaging from January 2012 to September 2015.

The patients’ inclusion criteria were: age over 18 years and diagnosis of HCM confirmed on interpretable ECG and magnetic resonance imaging performed at the Imaging Sector of the IDPC. The exclusion criteria were: age < 18 years; ejection fraction lower than 50% on cardiac magnetic resonance imaging; resistant arterial hypertension; presence of coronary artery disease, characterized by a coronary lesion greater than 50% on angiography; presence of Chagas disease; previous diagnosis of amyloidosis; endomyocardial fibrosis; Fabry disease; presence of definitive pacemaker; and septal myectomy or alcoholization prior to cardiac magnetic resonance imaging.

The HCM database of the Cardiomyopathy Sector of the IDPC was analyzed, and the medical records of 112 patients meeting this study inclusion criteria, which were stored at the Sector of Medical File and Statistics (SAME) of the IDPC, were assessed. After reviewing the medical files, 44 patients were excluded from the study.

The ECGs previously performed for the outpatient clinic visit at the Cardiomyopathy Sector were reviewed by the chief of the Tele-Electrocardiography Sector of the IDPC, in accordance with the 2016 Brazilian Guidelines on ECG Analysis and Report.^7^ The ECG report comprised the following variables: rhythm, heart’s QRS axis, ventricular and atrial overloads, intraventricular blocks, presence of strain, R wave size (millimeters) in leads DI, V1, V5 and V6, and T wave size (millimeters) in leads D1, V5 and V6.

**Figure 1:**
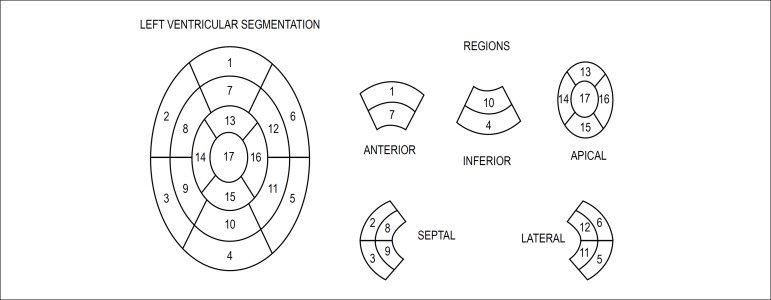
Left ventricular segmentation proposed by the American Heart Association.

Cardiac magnetic resonance imaging was assessed by the Radiology Team of the IDPC regarding the location of myocardial hypertrophy, based on the segmentation proposed by the American Heart Association,^8^ and the presence of delayed enhancement, as well as quantification of the fibrosis mass in grams. The 17 segments were grouped into five regions: anterior, inferior, lateral, septal and apical. (Figure 1)

**Figure 2:**
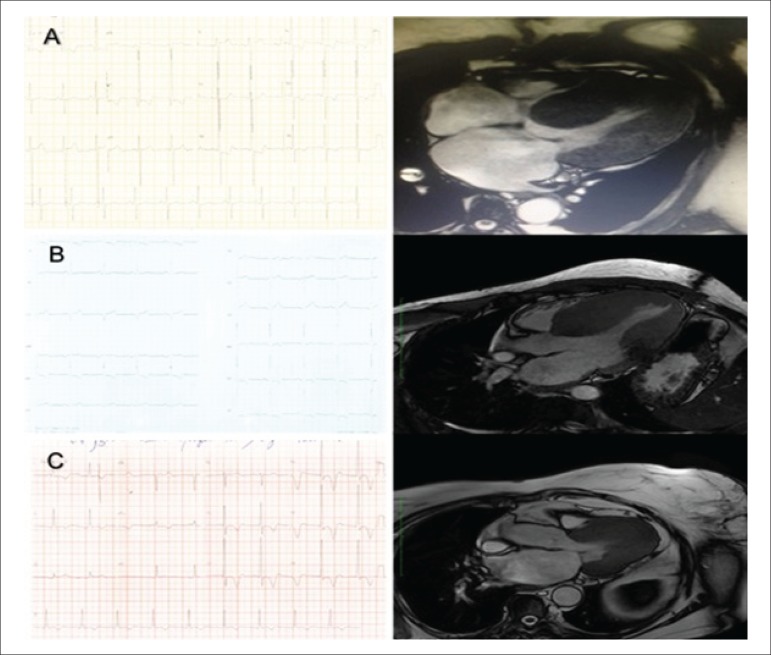
A) E.D.S., male sex, 35 years. ECG: R wave in D1 of 35 mm, and R wave in V5 of 29 mm. Magnetic resonance imaging compatible with concentric hypertrophy. Fibrosis mass of 128 g (greatest fibrosis mass found of all patients analyzed). B) M.L.S.F., female sex, 60 years. ECG: R wave in D1 of 23 mm, and R wave in V5 of 22 mm. Magnetic resonance imaging compatible with septal hypertrophy. C) P.M., male sex, 77 years. ECG: R wave of 35 mm in V5, and negative T wave of 12 mm with strain pattern. Magnetic resonance imaging compatible with apical hypertrophy.

Patients with hypertrophy > 15 mm in at least three of those regions were considered to have concentric hypertrophy. (Figure 2)

### Statistical analysis

The electrocardiographic variables previously described were compared with the region of hypertrophy, the presence of delayed enhancement and the amount of fibrosis identified on cardiac magnetic resonance imaging.

Normality of the data was assessed by use of Kolmogorov-Smirnov test, and nonparametric tests were used to compare between the groups. The summary measures median and 25th and 75th percentiles were calculated for the continuous variables, and nonparametric Kruskal-Wallis test was used to check the statistical significance between the groups, followed by two-by-two comparisons (Dunn’s multiple comparison test).

For attribute variables, the results were presented as percentages and frequency. Fisher-Freeman-Halton exact test was used to assess the statistical significance between the groups. Statistical significance level of 5% and 95% confidence interval were adopted.

The findings were recorded in an electronic spreadsheet of Microsoft Office Excel, version 2013e, and the Statistical Package for the Social Sciences (SPSS), version 21.0 for Windows®, was used for analysis.

## Results

**Figure 3:**
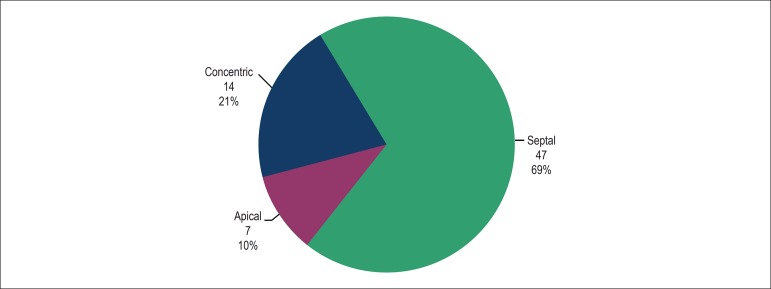
Distribution of the patients according to the hypertrophy pattern.

This study assessed 70 patients, 55.9% of the female sex, with a mean age of 51.3 years (ranging from 20 to 81 years). Of the six location patterns of hypertrophy, only three were found: apical (10%), concentric (21%) and septal (69%). (Figure 3)

**Figure 4:**
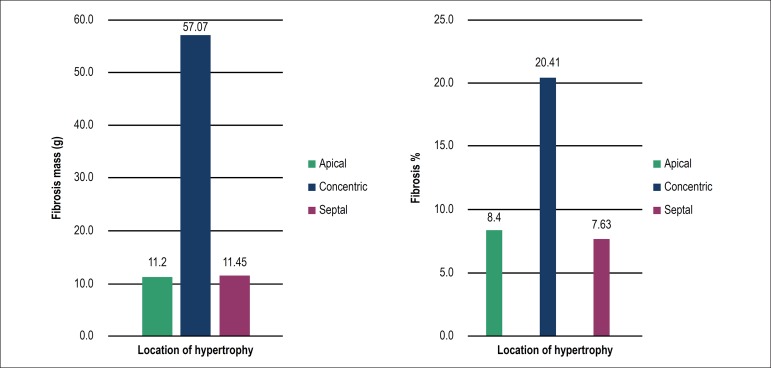
Means of the mass and percentage of myocardial fibrosis on cardiac magnetic resonance imaging according to the location of myocardial hypertrophy.

Most patients (81.4%) showed delayed enhancement on magnetic resonance imaging, and all patients with concentric hypertrophy had fibrosis. In quantifying the mass of fibrosis according to the location of hypertrophy, the highest mean (57.1 g) was observed in concentric hypertrophy as compared to the other locations (p = 0.001). (Table 1, Figure 4)

**Table 1:** Median and percentiles for mass and percentage of myocardial fibrosis on cardiac magnetic resonance imaging, according to the location of myocardial hypertrophy

Variables		Apical	Concentric	Septal	p-value			
					K-W	AxC	AxS	CxS
Fibrosis mass (grams)	Median (P25; P75)	0 (2; 27)	7 (40; 83.5)	0 (3.5; 15)	<.0001	0.0210	0.9974	< 0.0001
% Fibrosis	Median (P25; P75)	0 (2; 20)	4 (20; 31.75)	0 (3; 13)	0.0014	0.1160	0.9998	0.0010

P25: 25th Percentile; P75: 75th Percentile. P-values: K-W: Kruskal-Wallis test; multiple comparisons between the groups: AxC: Apical x Concentric; AxS: Apical x Septal; CxA: Concentric x Apical (Dunn's multiple comparison test).

The concomitant presence of right ventricular hypertrophy on magnetic resonance imaging was found in 35.7% of the patients with concentric hypertrophy, showing statistical significance (p = 0.0447) as compared to septal and apical hypertrophies.

Patients with apical hypertrophy more frequently had atrial fibrillation (14.3%), preserved heart axis being identified in 100% of the cases. Such findings, however, had no statistical significance (p = 0.7964, p = 0.6730, respectively).

Regarding ventricular overloads, there was higher prevalence of both left and right ventricular overloads (71.4% and 21.4%, respectively) in concentric hypertrophy, with no statistical significance (p = 0.1883, p = 0.2117, respectively).

The strain pattern showed statistical significance between the types of hypertrophy (p < 0.0001), being present in 100% of the apical hypertrophy cases and in 71.4% of the concentric hypertrophy cases.

Left atrial overload was more frequent in apical hypertrophy (42.9%), with no statistical significance (p = 0.4082). However, right atrial overload was rare, being identified in only two patients with septal hypertrophy.

**Figure 5:**
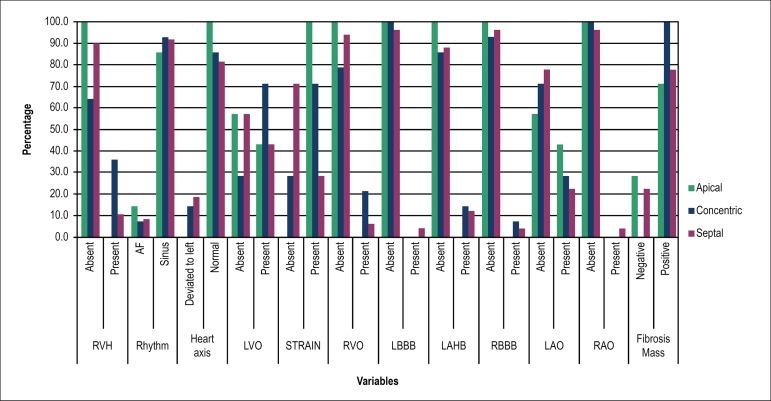
Percentages of the attribute variables according to location of myocardial hypertrophy. RVH: right ventricular hypertrophy; AF: atrial fibrillation; LVO: left ventricular overload; RVO: right ventricular overload; LBBB: left bundle-branch block; LAHB: left anterior hemiblock; RBBB: right bundle-branch block; LAO: left atrial overload; RAO: right atrial overload.

Intraventricular blocks, such as left bundle-branch block, right bundle-branch block and left anterior hemiblock, were infrequent in the three types of hypertrophy, with no statistical difference between the groups. (Table 2, Figure 5)

**Table 2:** Frequency and percentages for the attribute variables according to the location of myocardial hypertrophy

Variables	Group	Apical	Concentric	Septal	p-value
Right ventricular hypertrophy	Absent	7 (100.0%)	9 (64.3%)	44 (89.8%)	0.0447
	Present	0 (0.0%)	5 (35.7%)	5 (10.2%)	
Rhythm	AF	1 (14.3%)	1 (7.1%)	4 (8.2%)	0.7964
	Sinus	6 (85.7%)	13 (92.9%)	45 (91.8%)	
Heart axis	Deviated to left	0 (0.0%)	2 (14.3%)	9 (18.4%)	0.6730
	Normal	7 (100.0%)	12 (85.7%)	40 (81.6%)	
LVO	Absent	4 (57.1%)	4 (28.6%)	28 (57.1%)	0.1903
	Present	3 (42.9%)	10 (71.4%)	21 (42.9%)	
Strain	Absent	0 (0.0%)	4 (28.6%)	35 (71.4%)	< 0.0001
	Present	7 (100.0%)	10 (71.4%)	14 (28.6%)	
RVO	Absent	7 (100.0%)	11 (78.6%)	45 (93.8%)	0.1990
	Present	0 (0.0%)	3 (21.4%)	3 (6.3%)	
LBBB	Absent	7 (100.0%)	14 (100.0%)	47 (95.9%)	1.0000
	Present	0 (0.0%)	0 (0.0%)	2 (4.1%)	
LAHB	Absent	7 (100.0%)	12 (85.7%)	43 (87.8%)	0.8548
	Present	0 (0.0%)	2 (14.3%)	6 (12.2%)	
RBBB	Absent	7 (100.0%)	13 (92.9%)	47 (95.9%)	0.6634
	Present	0 (0.0%)	1 (7.1%)	2 (4.1%)	
LAO	Absent	4 (57.1%)	10 (71.4%)	38 (77.6%)	0.4615
	Present	3 (42.9%)	4 (28.6%)	11 (22.4%)	
RAO	Absent	7 (100.0%)	14 (100.0%)	47 (95.9%)	1.0000
	Present	0 (0.0%)	0 (0.0%)	2 (4.1%)	

AF: atrial fibrillation; LVO: left ventricular overload; RVO: right ventricular overload; LBBB: left bundle-branch block; LAHB: left anterior hemiblock; RBBB: right bundle-branch block; LAO: left atrial overload; RAO: right atrial overload. P-value for the Fisher-Freeman-Halton test.

The analysis of the size of the R wave in lead DI showed higher mean (15.6 mm) in concentric hypertrophy, and lower mean in septal hypertrophy (10 mm), with a significant difference between the three groups (p = 0.0280). In lead V1, the R wave showed no difference in its size (p = 0.9563).

As compared to septal and concentric hypertrophies, apical hypertrophy showed greater R wave amplitude in leads V5 and V6 (means of 26.9 mm and 26 mm, respectively), with statistical significance (p = 0.0391, p = 0.0148, respectively).

Regarding ventricular repolarization, apical hypertrophy correlated with the highest T wave negativity in DI (-3.8 mm), V5 (-10.2 mm) and V6 (-7.9 mm), with statistical significance in the three leads (p < 0.001). (Table 3)

**Table 3:** Median and percentiles of the continuous variables according to location of myocardial hypertrophy

Variables	Group	Apical	Concentric	Septal	p-value			
					K-W	AxC	AxS	CxS
R D1 (mm)	Median (P25, P75)	9 (11; 13)	9 (14; 19.5)	5.75 (8.5; 13)	0.0280	0.6870	0.2717	0.0444
R V1 (mm)	Median Median (P25, P75)	0 (1.5; 6)	0.88 (1.5; 4.13)	0 .88 (1.75; 3.25)	0.9563			
R V5 (mm)	Median (P25, P75)	20 (22; 35)	12 (21.5; 27)	9 (15; 22.25)	0.0391	0.5481	0.0440	0.3785
R V6 (mm)	Median (P25, P75)	20 (25; 31)	9.75 (19; 21.75)	8.25 (13; 22)	0.0148	0.1577	0.0125	0.5619
T D1 (mm)	Median (P25, P75)	-4 (-3.5; -2)	-5.13 (-2.75; -1.88)	-2 (0; 2)	< 0.0001	0.9725	0.0032	0.0010
T V5 (mm)	Median (P25, P75)	-12 (-8; -6)	-6.63 (-4.5; -2)	-2.5 (2; 3.5)	< 0.0001	0.0487	0.0009	0.0040
T V6 (mm)	Median (P25, P75)	-9 (-7; -4)	-6 (-3; -2.5)	-3 (1; 2.5)	< 0.0001	0.0685	0.0016	0.0072

P25: 25th Percentile; P75: 75th Percentile. P-values: K-W: Kruskal-Wallis test; multiple comparisons between the groups: AxC: Apical x Concentric; AxS: Apical x Septal; CxA: Concentric x Apical (Dunn's multiple comparison test).

## Discussion

Analysis of the patients with HCM showed a mild predominance of the female sex (55.9%), which differs from that reported in other studies.^2^

The distribution of myocardial hypertrophy found in this study by use of magnetic resonance imaging showed predominance of the septal location of hypertrophy (69% of the cases), followed by the concentric (21%) and apical (10%) locations, similar to that reported in the literature.^9^ Mid-ventricular and lateral involvements, not identified in this study, are rare, with reported prevalence of 1% to 2% of the cases.^10^

The presence of delayed enhancement on cardiac magnetic resonance worsens the prognosis of patients with HCM. Moon JC et al.,^11^ in a prospective study with 53 patients with HCM undergoing magnetic resonance imaging with gadolinium, have concluded that the presence of fibrosis relates to the occurrence of ventricular arrhythmias, ventricular dilatation and sudden death. Concentric hypertrophy showed a bigger mass of fibrosis as compared to that of the other hypertrophy locations. The R wave amplitude in DI was higher in concentric hypertrophy, showing a possible electrocardiographic pattern correlated with that location.

However, in leads V5 and V6, the R wave amplitude measured in millimeters showed a significant correlation with apical hypertrophy, in accordance with the findings of other studies.^12^ The mean R wave amplitude in V5 and V6 in the apical region was 26 mm, which is similar to that described by Yamaguchi et al. in patients with apical hypertrophy confirmed on the echocardiogram.^12^ The analysis of the R wave in V1 failed to show a good correlation with the anatomic pattern of hypertrophy.

In addition, apical hypertrophy was related to higher T wave negativity in the leads DI, V5 and V6 (means of -3.8 mm, -10.2 mm, and -7.9 mm, respectively). Chen X et al.,^13^ assessing 118 patients with HCM, have observed that negative T waves associated with apical hypertrophy (p = 0.009), corroborating the present study. Giant T waves, described as inversion ≥ 10 mm in any anterior lead, were also associated with apical hypertrophy, being found in the patients of that study in leads V5 and V6.^14,15^ The same relationship has been reported by Song et al.,^15^ studying 70 patients, who have evidenced a correlation of a deep negative T wave with apical hypertrophy on magnetic resonance imaging (p = 0.018).

Regarding the analysis of the strain pattern on ECG (change in the ST segment and T wave), that electrocardiographic finding showed a 100% correlation with the anatomic location of apical hypertrophy of the left ventricle. In patients with concentric hypertrophy, that pattern of ventricular repolarization change was found in 71% of the cases, while in the septal pattern, only in 28% of the cases, with statistical significance. Sung-Hwan Kim et al.,^16^ analyzing 864 patients with HCM, have found that same correlation of the strain pattern with apical hypertrophy; however, that was assessed by use of echocardiography (p < 0.001). The specific analysis of that electrocardiographic finding and its correlation with magnetic resonance imaging findings in HCM have not been found in the literature.

Electrocardiographic left ventricular overload was more frequently found in patients with the concentric pattern of hypertrophy (71%) than in the others (42%), but there was no statistical significance in those correlations. Previous studies have only assessed the presence or absence of electrocardiographic criteria of left ventricular overload, without comparing that finding with the location of hypertrophy.^6,17^

## Conclusion

The importance of the ECG as an initial tool to assess patients with HCM is confirmed in this study, which evidenced electrocardiographic patterns that correlate with the location of hypertrophy on magnetic resonance imaging.

The greater amplitude of the R wave in the leads V5 and V6, and the inversion of the T wave in the leads DI, V5 and V6, already reported in previous studies, were significantly related to apical hypertrophy. The presence of the strain pattern on ECG, when HCM is suspected, suggests apical hypertrophy on magnetic resonance imaging.

Concentric hypertrophy was associated with wide R waves in DI and a greater mass of fibrosis on magnetic resonance imaging assessment.
